# Clinical features of right‐to‐left shunt in the different subgroups of migraine

**DOI:** 10.1002/brb3.1553

**Published:** 2020-02-03

**Authors:** Yanyan Ling, Min Wang, Xudong Pan, Hongqin Zhao

**Affiliations:** ^1^ Department of Neurology The Affiliated Hospital of Qingdao University Qingdao University Qingdao China

**Keywords:** migraine, right‐to‐left shunt, transcranial Doppler

## Abstract

**Objective:**

Several investigations have documented an association between migraine and right‐to‐left shunt (RLS). However, whether there are specific clinical features that can distinguish between migraine patients with and without RLS is unclear. This study aims to explore whether there are specific clinical features that can distinguish between migraine patients with and without RLS, and to investigate the relationship between the degree of shunt and clinical parameters of headache.

**Methods:**

In this study, we enrolled consecutive migraineurs who underwent a structured, standardized questionnaire for family and personal history and for detailed migraine features. RLS was diagnosed based on a contrast enhancement transcranial Doppler (c‐TCD) examination.

**Results:**

Overall, 113 migraine with aura (MA) and 192 migraine without aura (MO) patients were included. Patients with MA and RLS (MARLS+) had a higher frequency for sensory aura symptoms than those with MA without RLS (MARLS−) (27.4% vs. 10.0%, *p* = .03). Patients with MO and RLS (MORLS+) presented with significantly younger initial age of migraine onset and experienced more severe pain intensity than those with MO without RLS (MORLS−) (mean ± *SD*, 25.6 ± 8.9 vs. 29.8 ± 12.7 years, *p* = .008 and 5.9 ± 1.4 vs. 5.3 ± 1.3, *p* = .006, respectively). There was no relationship between the degree of shunt and the clinical parameters of headache.

**Conclusions:**

Our results indicate that MO patients presented with a younger initial age of migraine onset and that sensory aura symptoms in MA patients may predict the presence of RLS. However, we did not find support for relationship between the degree of shunt and clinical parameters of headache.

## Graphical abstract

The MARLS+ patients had a significant higher frequency for sensory aura symptom than MARLS− patients. The MORLS+ patients were significantly younger in the initial age of migraine onset, and more severe in the pain intensity than that in MORLS− patients. There was no relationship between the degree of shunt and the clinical parameters of headache.

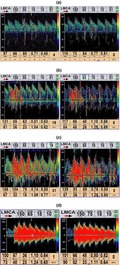

## 1 INTRODUCTION

There is a close association between migraine and right‐to‐left shunt (RLS), usually due to the presence of patent foramen ovale (PFO) (Ailani, 2014; Wang et al., 2018). Some studies have reported that the prevalence of RLS in migraine patients with aura (MA) and those without aura (MO) is 2.5‐fold and 1.4‐fold higher, respectively, than that found in healthy control subjects (Schwerzmann et al., 2005; Wang et al., 2018). A larger RLS is more common in MA and MO patients than in controls. Several investigations have documented that the closure of RLS brings relief to migraineurs and suggested that RLS may play an important role in triggering migraine attacks (Wahl et al., 2010). Nevertheless, how to distinguish migraine patients with and without RLS according to differences in their clinical features is still unknown.

This study aims to explore whether specific clinical features can differentiate between migraine patients with and without RLS in clinical practice and evaluate the relationship between the degree of RLS and clinical parameters of migraine.

## 2 METHODS

### 2.1 Study population

We enrolled consecutive patients diagnosed with migraine (age: 18–65 years) at the outpatient clinic of the Affiliated Hospital of Qingdao University from June 2016 to March 2019. Migraine was diagnosed according to the diagnostic criteria of the International Classification of Headache Disorders III‐beta (Headache Classification Committee of the International Headache Society (IHS), 2013). All participants were systematically examined by a contrast enhancement transcranial Doppler ultrasound (c‐TCD), brain magnetic resonance imaging (MRI), carotid ultrasound, and received physical and neurological examinations. Patients with intracranial or extracranial arterial stenosis or those with other central nervous system diseases—for example, cerebral hemorrhage, cerebral vascular malformations, and brain tumors—were rejected from this study based on our exclusion criteria. We also excluded those participants who did not have an adequate acoustic window and were unable to perform an effective Valsalva maneuver (VM).

The study was approved by the Ethics Committee of the Affiliated Hospital of Qingdao University. All study participants signed the informed consent form.

### 2.2 Assessment of the clinical characteristics of migraine and risk factors of cerebral vascular disease

Before TCD examination, the clinical features of migraine and risk factors of cerebral vascular disease were obtained from each patient, based on a questionnaire. Clinical characteristics of migraine including the initial age of migraine onset, mean monthly frequency of migraine attacks, duration of the headache phase (calculated as the time from the occurrence of pain to when either the pain dissipates or the patient falls asleep), and headache intensity (measured on a numeric rating scale (NRS), from 0 = no pain to 10 = maximal pain). The three aura characteristics included in this study were visual aura, unilateral somatic sensory troubles, and lateralized motor troubles occurring either before or during a headache. The cerebrovascular risk factors included hypertension, diabetes mellitus, cigarette smoking (including former smokers who had stopped smoking up to 6 months before the study), alcohol drinking, and hypercholesterolemia.

### 2.3 RLS detection

**Figure 1:**
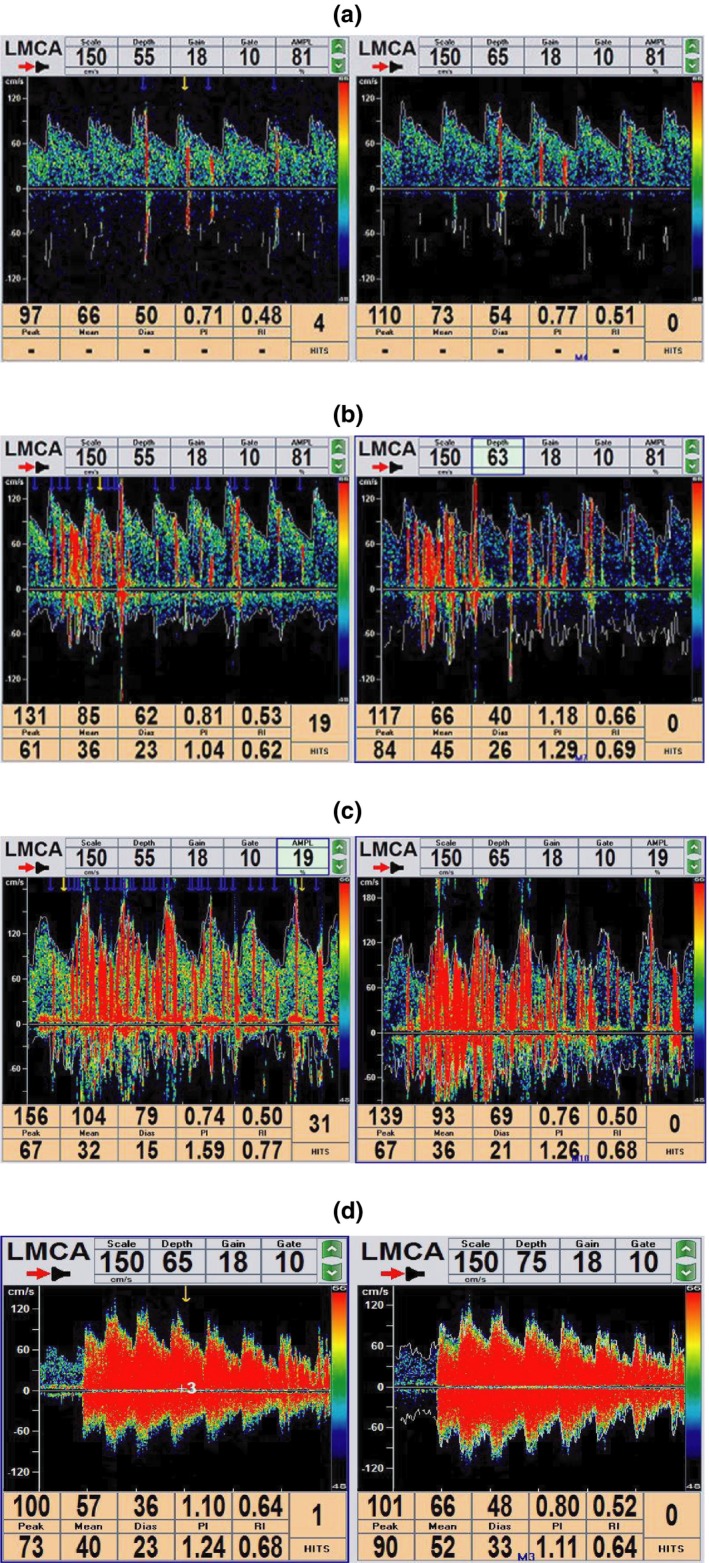
Three grades of shunt classification according to the microbubble (MB) counts in the Doppler spectrum *Note*. mild shunt = 1–10 MBs (a), moderate shunt =10–25 MBs (b), and large shunt= >25 MBs with shower (c) or curtain patterns (d)

We used a TCD detector (EMS‐9EB; Delica, China) to perform TCD examinations. We instructed each patient to take the supine position and used a 2‐Hz probe to monitor their left middle cerebral artery (MCA). An experienced nurse inserted a 20G casing needle into the patient's left elbow vein, and two 20 ml syringes were connected to the needle by means of a triple link system. A mixture consisting of 9 ml saline, 1 ml air, and 1 drop of the patient's elbow vein blood was exchanged between the two syringes 20 times, and a homogeneous contrast agent was quickly passed through the elbow vein. We conducted the procedure three times: the first time during normal breathing and two more times during the VM procedure. The efficacy of the VM was identified by a 25% reduction in peak systolic velocity in the MCA. VM was started 5 s after starting the injection and lasted for 10 s. The overall duration of VM therefore corresponded to 15 s after the starting of the injection (Jauss & Zanette, 2000), with each interval lasting at least 5 min from the last observed microbubble (MB). RLS was diagnosed based on the presence of at least one MB in the 20 s following the initiation of the procedure. We assessed the degree of shunt based on the amount of MBs detected in the Doppler spectrum using unilateral MCA monitoring as follows: mild shunt (1–10 MBs), moderate shunt (10–25 MBs), and large shunt (>25 MBs with shower or curtain patterns) (Wang et al., 2018) (Figure 1). This assessment was performed by an experienced doctor who was blind to the patients' diagnoses.

### 2.4 Statistical analysis

The statistical analyses were carried out using the SPSS 24.0 software (IBM Corp., 2016. Armonk, NY, USA). Continuous variables were analyzed by Student's *t* test, and intergroup differences between nominal variables were tested by the chi‐square test or Fisher's exact test. The relationship between the degree of shunt and clinical parameters of headache was analyzed with ANOVA. All statistical tests were two‐sided, and *p* < .05 was considered as statistically significant.

## 3 RESULTS

**Figure 2:**
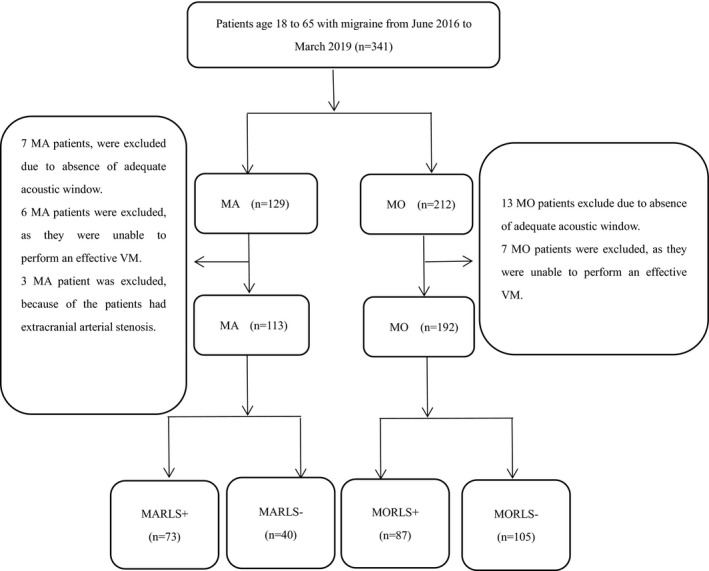
Flowcharts of patient selection Abbreviations: n: number of per clinical subgroup; %: percentage of patients per clinical subgroup; MA: migraine with aura; MO: migraine without aura; RLS+: presence of right‐to‐left shunt; RLS‐: absence of right‐to‐left shunt; MARLS+: patient with MA and RLS; MARLS‐: patient with MA without RLS; MORLS+: patient with MO and RLS; MORLS‐: patient with MO without RLS

A total of 341 patients were enrolled in this study and were divided into two subgroups: 129 patients in the MA subgroup and 212 patients in the MO subgroup. Twenty patients—including thirteen MO patients and seven MA patients—were excluded because of the absence of an adequate acoustic window. Seven MO patients and six MA patients were also excluded due to their inability to perform an effective VM. Three MA patients were excluded as they had extracranial arterial stenosis (Figure 2). Overall, 305 patients (mean ± *SD*, 38.9 ± 13.4 years) were included, including 113 MA patients (37.3 ± 14.0 years) and 192 MO patients (39.8 ± 13.0 years). In the MA subgroup, 73 (64.6%, 73/113) patients had RLS, consisting of 25 (34.2%, 25/73) mild shunt patients, 8 (11.0%, 8/73) moderate shunt patients, and 40 (54.8%, 73/113) large shunt patients. In the MO subgroup, the prevalence of RLS was 45.3% (87/192); of the 87 patients, 45 (51.7%, 45/87) had a mild shunt, 6 (6.9%, 6/87) had a moderate shunt, and 36 (41.4%, 36/87) patients had a large shunt (Table 1).

**Table 1:** Prevalence of the three grades of shunt in the MA and MO groups

Groups	Overall prevalence of RLS%, (*n*)	Mild shunt %, (*n*)	Moderate shunt %, (*n*)	Large shunt %, (*n*)
MA	64.6% (73/113)	34.2% (25/73)	11.0% (8/73)	54.8% (40/73)
MO	45.3% (87/192)	51.7% (45/87)	6.9% (6/87)	41.4% (36/87)

Note

mild shunt = 1–10 MBs, moderate shunt = 10–25 MBs, and large shunt = >25 MBs with shower or curtain patterns.

Abbreviations: MA, migraine with aura; MO, migraine without aura; RLS, right‐to‐left shunt.

### 3.1 Differences between the MA with and without RLS groups

The 113 MA patients were divided into two groups, with 73 (64.6%) patients in the MA with RLS (MARLS+) group and 40 (35.4%) patients in the MA without RLS (MARLS−) group. There was no significant difference in age (37.7 ± 14.8 vs. 36.6 ± 12.4 years, *p* = .4) or gender (females: 80.8% vs. 67.5%, *p* = .2) between the two groups. There was a significant difference in the sensory aura symptoms of the MARLS+ and MARLS− groups (27.4% vs. 10.0%, *p* = .03). However, the clinical characteristics of MA—that is, the visual aura, motor aura, initial age of migraine onset, pain intensity, mean monthly frequency of migraine attacks, and duration of migraine pain—were not significantly different (*p* > .05) between the groups. Moreover, there were no significant differences in the risk factors of cerebral vascular disease—that is, hypertension, diabetes mellitus, cigarette smoking, alcohol drinking, and hypercholesterolemia—between the groups (Table 2).

**Table 2:** Clinical characteristics of migraine and risk factors of cerebral vascular disease in MA and MO groups, subdivided by presence of RLS

Variables	MA		*p*‐Value	MO		*p*‐Value
	RLS+ *n* = 73	RLS− *n* = 40		RLS+ *n* = 87	RLS− *n* = 105	
Age, (years, mean ± *SD*)	37.7 ± 14.8	36.6 ± 12.4	.4	40.6 ± 12.4	39.2 ± 13.5	.5
Sex (female), *n* (%)	59 (80.8%)	27 (67.5%)	.2	65 (74.7%)	71 (67.6%)	.2
Smoking, *n* (%)	5 (6.8%)	3 (7.5%)	1.0	9 (10.3%)	7 (6.7%)	.8
Alcohol drinking, *n* (%)	4 (5.5%)	6 (15.0%)	.2	9 (10.3%)	8 (7.6%)	.8
Hypertension, *n* (%)	7 (12.3%)	1 (2.5%)	.3	5 (5.7%)	6 (5.7%)	1.0
Diabetes, *n* (%)	4 (5.5%)	1 (2.5%)	.3	2 (2.3%)	2 (1.9%)	1.0
Hyperlipemia, *n *(%)	7 (9.6%)	3 (7.5%)	1.0	5 (5.7%)	4 (3.8%)	.7
Initial age of migraine onset (years, mean ± *SD*)	25.9 ± 12.7	29.5 ± 14.1	.2	25.6 ± 8.9	29.8 ± 12.7	.008
Family history of migraine, *n* (%)	15 (20.6%)	4 (10.0%)	.2	14 (16.1%)	30 (28.6%)	.9
Pain frequency (mean ± *SD*)	15 (20.5%)	4 (10.0%)	1.0	5.1 ± 4.7	4.2 ± 3.6	.2
Pain intensity (mean ± *SD*)	15 (20.5%)	4 (10.0%)	.3	5.9 ± 1.4	5.3 ± 1.3	.006
Pain duration (mean ± *SD*)	9.7 ± 7.5	13.3 ± 18.2	.2	10.9 ± 7.7	11.1 ± 9.4	.9
Visual aura, *n* (%)	50 (68.5%)	33 (82.5%)	.1			
Sensory aura, *n* (%)	20 (27.4%)	4 (10.0%)	.03			
Motor aura, *n* (%)	3 (4.1%)	3 (7.5%)	.7			

Note

Frequency: mean monthly frequency of migraine attacks (mean ± *SD*). Pain intensity was measured using a numeric rating scale (NRS; from 0 = no pain to 10 = maximal pain; mean ± *SD*). Migraine duration: calculated from the time of occurrence of pain to the time when either the pain dissipates or the patient falls asleep (hr; mean ± *SD*).

Abbreviations: MA, migraine with aura; MO, migraine without aura; RLS+, presence of right‐to‐left shunt; RLS−, absence of right‐to‐left shunt.

### 3.2 Differences between the MO with and without RLS groups

Of the 192 MO patients, 87 (45.3%) were in the MO with RLS (MORLS+) group and 105 (54.7%) were in the MO without RLS (MORLS−) group. There were no obvious intergroup differences in age (40.6 ± 12.4 vs. 39.2 ± 13.5 years, *p* = .5) or gender (female: 74.7% vs. 67.6%, *p* = .2). However, patients in the MORLS+ group were significantly younger at the initial age of migraine onset (25.6 ± 8.9 vs. 29.8 ± 12.7 years, *p* = .008), and experienced higher pain intensity (5.9 ± 1.4 vs. 5.3 ± 1.3; *p* = .006) than the MORLS‐ group patients. However, there were no significant differences between the MORLS+ and MORLS− groups in terms of their cerebrovascular risk factors or other clinical characteristics of migraine (Table 2).

### 3.3 Clinical parameters of MA in the mild, moderate, and large shunt groups

There were no significant differences between the mild, moderate, and large shunt groups in terms of age (32.5 ± 13.5, 31.5 ± 8.1, and 38.7 ± 12.7 years, respectively; *p* = .1) or gender (female: 93.3%, 66.7%, and 80.8%, respectively; *p* = .1). In addition, there were no statistically significant differences in the clinical parameters of headache including the pain intensity, initial age of migraine onset, pain duration, and mean monthly frequency of migraine attacks (all *p* > .05, Table 3).

**Table 3:** Clinical parameters of migraine with aura in the mild, moderate, and large shunt groups

**Variables**	Mild shunt *n* = 25	Moderate shunt *n* = 8	Large shunt *n* = 40	*p*‐Value
Initial age of migraine onset (years, mean ± *SD*)	24.4 ± 13.9	23.9 ± 8.0	26.4 ± 10.8	.4
Frequency of migraine attacks (mean ± *SD*)	3.5 ± 2.2	3.5 ± 2.7	5.0 ± 4.3	.2
Pain intensity (mean ± *SD*)	5.4 ± 1.5	5.5 ± 0.5	5.9 ± 1.4	.2
Pain duration (hr, mean ± *SD*)	9.1 ± 6.2	8.9 ± 3.7	6.0 ± 1.5	.8

Note

Mild shunt = 1–10 MBs, moderate shunt = 10–25 MBs, and large shunt = >25 MBs with shower or curtain patterns.

### 3.4 Clinical parameters of MO in the mild, moderate, and large shunt groups

There were no significant differences in age (42.3 ± 12.4, 32.2 ± 1.6, and 39.8 ± 12.9 years, respectively; *p* = .2), gender (female: 82.2%, 66.7%, and 66.7%, respectively; *p* = .3), initial age of migraine onset, mean monthly frequency of migraine, pain intensity, or pain duration among the three groups (all *p* > .05, Table 4).

**Table 4:** Clinical parameters of migraine without aura in the mild, moderate, and large shunt groups

Variables	Mild shunt *n* = 45	Moderate shunt *n* = 6	Large shunt *n* = 36	*p*‐Value
Initial age of migraine onset (years, mean ± *SD*)	25.6 ± 9.4	24.3 ± 4.2	25.7 ± 9.1	.9
Frequency of migraine attacks (mean ± *SD*)	4.8 ± 4.7	4.7 ± 1.7	5. ± 5.2	.8
Pain intensity (mean ± *SD*)	5.8 ± 1.3	6.0 ± 1.3	5.9 ± 1.5	.8
Pain duration (hr, mean ± *SD*)	11.4 ± 10.4	6.9 ± 3.9	11.3 ± 6.9	.4

Note

Mild shunt = 1–10 MBs, moderate shunt = 10–25 MBs, and large shunt = >25 MBs with shower or curtain patterns.

## 4 DISCUSSION

Previous studies have reported an association between migraine and RLS, with a higher prevalence of migraine in patients with RLS, and a high prevalence of RLS in patients with migraine (Ailani, 2014; Azarbal et al., 2005; Garg et al., 2010). This phenomenon might imply that the RLS plays pivotal role in migraines (Anzola et al., 2008).

In the MA subgroup, we observed that the MARLS+ patients had a higher frequency of sensory aura symptoms than in the MARLS− group. Anzola et al. also reported similar results in MA patients. In addition, they also found that MARLS+ patients were significantly younger than MARLS− patients; however, we did not note this in our study (Anzola et al., 2008).

In the MO subgroup, MORLS+ patients were significantly younger than MORLS− patients at the initial age of migraine onset and experienced higher migraine pain intensity. Their younger age at migraine onset might be due to the long‐standing nature of the shunt. In a retrospective study, Dalla Volta et al. could not find any difference in clinical features between migraine patients (both MA and MO) with and without RLS (Dalla Volta et al., 2005). However, in a recent study, Claudia et al. found that MARLS+ patients presented with a younger age of migraine onset than MARLS− patients (Altamura et al., 2019).

We found no association between the degree of RLS and clinical parameters of headache in both the MA and MO subgroups in this study. Likewise, Garg et al. (2010) also found no association between the degree of RLS and clinical features of migraine headaches. However, He et al. (2019) reported that the headache frequency, migraine disability assessment score, and headache impact test‐6 score were significantly higher in patients with RLS than those without RLS. The degree of shunt does not appear to be related to the clinical parameters of headache, suggesting that the mechanism of the association between RLS and migraine is not only related to the degree of shunt, but also to the shear stress. Sadrameli, Gadhia, Kabir, and Volpi (2018) hypothesized that a larger degree of RLS transfers more chemical triggers, paradoxical microemboli into the systemic circulation, thus causing migraine attacks, while a small degree of shunting is more likely to activate platelets attributed to shear stress, which releases serotonin and induces migraine attacks when the blood passes through the intracardiac shunt.

Several theories have been proposed for the potential mechanism of association between migraine and RLS. The chemical triggers of migraine (such as serotonin), while normally inactivated by pulmonary circulation (Borgdorff & Tangelder, 2012; Hildick‐Smith & Williams, 2017), can bypass the pulmonary circulation filter through the RLS and enter cerebral circulation, leading to migraine seizures. Serotonin could also be released from platelets activated by shear stress, ultimately leading to migraine attacks, and this process may be initiated by the presence of a PFO (Sadrameli et al., 2018).

Paradoxical microembolism has also been suggested as a migraine trigger. Microemboli—such as small venous blood clots and platelet aggregations—are generally trapped in the lungs and can act as potential triggers for migraines when introduced into the arterial circulation through RLS (Fuller & Jesurum, 2009). In addition, animal experiments have shown that microemboli can cause migraine attacks via the induction of cortical spreading depression (CSD) (Lauritzen, 1994).

Transient hypoxic episodes—which occur when deoxygenated venous blood enters and combines with oxygenated arterial blood via the RLS—may also explain the mechanism of association between migraine and RLS (Mojadidi et al., 2019; Tobis & Azarbal, 2005). Moreover, hypoxemia has also been shown to be a potential trigger of CSD (Hildick‐Smith & Williams, 2017).

Percutaneous closure of the RLS has recently been proposed as an effective treatment for migraine attacks. Wilmshurst et al. first reported an improvement in migraine symptoms after percutaneous transcatheter closure of the RLS (Wilmshurst, Nightingale, Walsh, & Morrison, 2000). Several subsequent observational, nonrandomized trials, conducted globally, have shown that migraine symptoms are improved among migraineurs after percutaneous closure of the PFO (Gupta, 2004; Wahl et al., 2010). In recent years, a meta‐analysis of randomized clinical trials—including three trials consisting of 448 total patients (average follow‐up of 10 months)—has shown that between the PFO closure and control groups, there was no significant difference among subjects who experienced a complete cessation of migraine attacks. However, the PFO closure group had significantly fewer migraine attacks and total migraine days per month on average (Kheiri et al., 2018).

Some studies have suggested that antiplatelet drug treatment is effective in preventing RLS‐related migraine attacks (Spencer, Qureshi, & Sommer, 2014). Serotonin can bypass the lungs and reach the cerebral circulation, thus triggering migraine symptoms in patients with RLS. Antiplatelet drugs, by inhibiting the release of serotonin, could potentially reduce the cerebral concentrations of serotonin, thus reducing the frequency of migraines (Wilmshurst, Nightingale, Walsh, & Morrison, 2005).

A part of the migraineurs patient population may present with RLS. Migraine patients are at a higher risk of stroke, at least partly due to the high prevalence of RLS in these patients (Altamura et al., 2019). However, the most recent European consensus does not recommend screening for RLS in all migraine patients (Mitsikostas et al., 2015). Therefore, it is critical to identify migraine features that can potentially predict RLS, in order to help doctors select migraine patients with RLS. For migraine patients with RLS, it may be beneficial to choose additional treatments—such as antiplatelet drugs or percutaneous closure of the PFO to prevent migraine‐related stroke.

There are some limitations in this study. First, this is a single‐center study. Second, we used TCD examination to detect RLS, including intracardiac and extracardiac shunts. However, we could not identify the exact location of the RLS using this technique.

## 5 CONCLUSION

In clinical practice, migraineurs may be advised to accept RLS examination when they manifest a younger initial age of migraine onset or are accompanied with sensory aura symptoms. However, these findings do not support an association between the degree of shunt and clinical parameters of headache.

## CONFLICT OF INTEREST

The authors declare no potential conflicts of interest with respect to the research, authorship, and/or publication of this article.

## Data Availability

The data that support the findings of this study are available from the corresponding author upon reasonable request.
